# Fibrous Polymer-Based Composites Obtained by Electrospinning for Bone Tissue Engineering

**DOI:** 10.3390/polym14010096

**Published:** 2021-12-28

**Authors:** Kristina Peranidze, Tatiana V. Safronova, Nataliya R. Kildeeva

**Affiliations:** 1 Department of Materials Science, Lomonosov Moscow State University, Leninskie Gory 1, 119991 Moscow, Russia; t3470641@yandex.ru; 2 Department of Chemistry, Lomonosov Moscow State University, Leninskie Gory 1, 119991 Moscow, Russia; 3 Department of Chemistry and Technology of Polymer Materials and Nanocomposites, The Kosygin State University of Russia, Malaya Kaluzhskaya 1, 119071 Moscow, Russia; kildeeva@mail.ru

**Keywords:** polymer scaffolds, bone tissue engineering, electrospinning, nanofibers, extracellular matrix

## Abstract

Currently, the significantly developing fields of tissue engineering related to the fabrication of polymer-based materials that possess microenvironments suitable to provide cell attachment and promote cell differentiation and proliferation involve various materials and approaches. Biomimicking approach in tissue engineering is aimed at the development of a highly biocompatible and bioactive material that would most accurately imitate the structural features of the native extracellular matrix consisting of specially arranged fibrous constructions. For this reason, the present research is devoted to the discussion of promising fibrous materials for bone tissue regeneration obtained by electrospinning techniques. In this brief review, we focus on the recently presented natural and synthetic polymers, as well as their combinations with each other and with bioactive inorganic incorporations in order to form composite electrospun scaffolds. The application of several electrospinning techniques in relation to a number of polymers is touched upon. Additionally, the efficiency of nanofibrous composite materials intended for use in bone tissue engineering is discussed based on biological activity and physiochemical characteristics.

## 1. Introduction

Tissue engineering is a rapidly evolving field of regenerative medicine that originates from a scientific branch related to biomaterials development and incorporates such disciplines as cell biology, materials science, and mechanics. The main objectives of tissue engineering are focused on tissue recovery, replacement, or regeneration via combining certain interdisciplinary approaches to the manufacturing of a material with strictly defined physical and chemical properties. Over the past few decades, an approach aimed at formation of new tissues with required functions by isolation and expansion of target donor cells in vitro for their further seeding and growth in implanted constructions has become widespread, and received more study against the background of other ways to eliminate tissue damages [1]. Currently, the use of such a cellular approach in relation to bone, cartilage, tendon, and ligament tissues, among others, demonstrates moderate success [2]. However, modern materials and techniques for their manufacturing still do not meet all the necessary requirements to be applied in the field of regenerative medicine.

A tremendous number of studies on the development of biomaterials with designable milieus devoted to the investigation of interactions between donor stem cells and materials microenvironment showed that scaffolds mimicking the extracellular matrix (ECM) in structure and properties held much promise in tissue engineering. Ideally, the engineered scaffold should possess the functional properties of ECM’s native components of the tissue to be regenerated [3]. According to the above-mentioned, the majority of matrices intended for use as scaffolds in regenerative medicine are often made of biocompatible biodegradable components of a given chemical and phase composition with the surface topography that provides cell attachment, proliferation, and migration. If necessary, the surface of the materials should be suitable for functionalization with medical and/or antibacterial drugs promoting tissue repair. In the case of dense tissues, such as bone and cartilage, particular attention is paid to strength characteristics while maintaining flexibility and permeable architecture. To date, a great variety of polymer-based biomaterials, including composite hydrogels obtained in the form of membranes, fibers, and complex 3D-structures have been researched.

**Figure 1:**
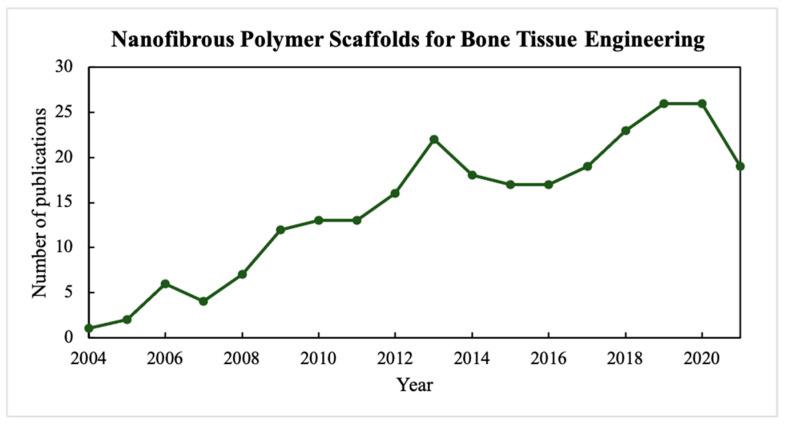
The number of published papers on nanofibrous polymer scaffolds for bone tissue engineering.

Fibrous polymer scaffolds attract a lot of attention due to the high affinity of their structure with the structure of native ECM that consists of nano-/microscale protein fibers with various arrangements. In addition, compared to 3D polymer hydrogels, which often lack the native ECM’s anisotropy type, synthetic fibrous constructions possess higher surface to volume ratio that is similar to crosslinked collagen nanofibers forming the ECM’s basis [4]. Hence, the main research efforts are directed to the improvement of nanoscale fibrous biomaterials to replicate the natural matrix structure and morphology, and provide a comfortable microenvironment for implanted cells. Based on Scopus data, scientists have made more and more attempts to develop a suitable fibrous matrix for use in bone tissue engineering. As shown in Figure 1 (keywords = “nanofibrous polymer scaffolds for bone tissue engineering”), the number of publications on fibrous polymer scaffolds for bone regeneration has increased significantly, which indicates the prospects of this research area. The fiber manufacturing occurs via various capillary and capillary-free electrospinning techniques [5]. Moreover, a large number of research sources provides methods for obtaining electrospun composite hydrogels made up of two-, three-component polymer solutions, or composite polymer hydrogels filled with bioactive mineral inclusions to promote certain mimicry type.

The review of electrospun materials from different areas of tissue engineering appears to be a rather laborious task, and involves a multitude of significant classifications of polymer materials and comparative characteristics of their advantages and drawbacks while using a specific electrospinning type. In this brief review, we propose to focus on the study of modern materials and electrospinning techniques for the development of polymer-based fibrous bioscaffolds for bone tissue engineering (BTE). In order to emphasize the most prospective achievements in BTE in the last five years, the present article summarizes the recent approaches to design fibrous scaffolds via electrospinning. It starts with a short classification of composite polymer hydrogels intended to form artificial matrix. Subsequently, the key fiber preparation techniques and their limitations are discussed in accordance with the specified classification. Based on the presented scaffolds functional properties, we give a brief assessment regarding the effectiveness of materials application to BTE, and provide the outlook for future research.

## 2. Modern Polymer-Based Fibrous Composites

### 2.1. Polymers for ECM Imitation in BTE

Based on recent research papers in the field of polymer electrospun scaffolds for BTE, it undoubtedly follows that a substantial number of natural and synthetic polymers in pure or modified forms in combination with other polymers, crosslinking agents, bioactive organic molecules, and mineral particles have already been discussed as promising components for the artificial matrix construction [6,7,8,9,10,11]. Many studies provide classifications of polymers according to their nature, molding ability, and, of course, biological properties such as controlled degradation rate and support of cell metabolic activity. Widely studied polymers intended to form artificial fibrous matrices are presented in Table 1, taking into account the features of molding process via electrospinning and the performance of positive interaction with the implanted cells. The simplest and most easy-to-understand classification covering the main types of modern polymer-based composite materials is presented in [12]. The authors propose to divide electrospun materials into five categories, including natural polymer-natural polymer, natural polymer-synthetic polymer, synthetic polymer-synthetic polymer, crosslinked polymers, and polymer-inorganic materials. In the present research, we suggest adhering to this classification and focusing on the most significant examples of polymer–polymer and polymer–inorganic fibrous materials that have proven themselves as advanced bioscaffolds with a vast surface area for cell attachments.

*Characteristics of the bone matrix*. The organic constituent of bone tissue is represented by non-mineralized ECM (osteoid) that consists predominantly of collagen type I, III, and V. Collagen-based matrix acts as a mechanical frame for bone cells providing vital mechanical support. Thus, macromolecules of collagen-1, the most significant fibrillar protein in the matrix, are characterized by a diameter of 80–100 nm, and its fibers are mainly located parallel to the direction of the most probable mechanical strain on the bone, which ensures tissue elasticity [13]. The fibrils of collagen type 1 assembled from triple polypeptide helices interact with collagenous and other proteins, which results in more complex fiber arrangement. Collagen types III and V present in smaller amounts and participate in fibrillogenesis of type I collagen, and inter- and intra-chain crosslinking of macromolecules. Other constituents of organic ECM include noncollagenous proteins, such as proteoglycans, glycoproteins, and small integrin-binding ligand N-linked glycoproteins (SIBLINGs), that play a key role in the regulation of osteoblast differentiation and initiation of ECM mineralization. Biomineralization is considered a core process that occurs during bone tissue formation. This process leads to the deposition of hydroxyapatite (Ca_10_(PO_4_)_6_(OH)_2_), the main inorganic component of hard tissues, and collagen produced during biomineralization performs the function of a template for mineral crystals deposition [14].

*Natural polymers*. The desire to imitate natural bone tissue to the greatest extent led to ubiquitous study of nanofibrous materials based on collagen and other natural polymers that are very similar to collagen-1 in chemical composition and structure. Thus, a large number of papers are devoted to the development of non-woven fibrous scaffolds based on gelatin [15], keratin [16], fibroin [17], and fibrin [18]. In particular, gelatin attracts research interest due to the fact that its materials contribute to cell adhesion and proliferation, which is explained by the presence of a peptide sequence l-arginine–aspartic acid in the structure. Special attention has been paid to osteogenic biomaterials based on silk fibroin protein that ensures high cellular compatibility and template the growth of hydroxyapatite (HAP) crystals [19]. Along with the specified polymers, chitosan [20], cellulose [21], alginate [22], and GelMA (polymer made of gelatin and methacrylate anhydride) [23] show a great potential for use in BTE when designing bioscaffolds. For instance, many papers discuss opportunities for obtaining electrospun chitosan-consisting materials. 

Chitosan formed by partial chitin deacetylation is a polysaccharide that possesses essential biocompatibility and pH-variable biodegradability, and considered a great wound healing promoter in regenerative medicine [38,39]. However, there exist specific technological difficulties associated with chitosan fiber manufacturing via electrospinning, since the molding process often requires the injection of toxic acidic reagents, such as trifluoroacetic, acetic, or hydrochloric acids [40,41], to enhance the stretching of the jet from high-molecular solutions. The solution to this key problem has been found by combining chitosan with the synthetic polymers (poly(vinyl alcohol) and poly(ethylene oxide)) with lower molecular weight and good molding ability conditioned by special conductivity and viscosity of their aqueous solutions [42,43,44]. In recent years, the general interest has grown in relation to GelMA that allows to produce stable crosslinked hydrogels using UV irradiation and photoinitiators. Moreover, the property of controllable degradation rate led to the development of GelMA-based complex fibrous structures to be placed into varied shaped bone defects [45]. The work [46] describes the approach to the development of pH-sensitive GelMA/f-CNOs-based hydrogels by using a photo-crosslinking method. γ-cyclodextrin/DOX-complexes encapsulated into the matrix with controlled release of DOX demonstrated not only a sufficient cell viability with human fibroblasts, but the improved mechanical and swelling properties. Thus, f-CNOs (poly-(*N*-(4-aminophenyl) methacrylamide)) carbon nano-onions) insertion led to the increased tensile strength (356.1 ± 3.4 MPa) and Young’s modulus (41.8 ± 1.4 GPa) values of supramolecular hydrogels. The enhanced drug release was detected from NCST3 system over 18 days at pH 4.5. All that makes GelMA/f-CNOs-based hydrogels promising for use as delivery systems for encapsulated therapeutic drug molecules.

From the analysis of the literature, it can be concluded that the combination of different natural polymers in order to manufacture composite electrospun nanofibers is a simple and effective approach to provide biocompatible substrates supporting vital cell activity. To date, despite a remarkable number of attempts to prepare a fibrous composite material consisting of collagen and other natural polymer, suitable artificial ECM of such phase composition has not been developed yet. The key obstacles to its development are related to weak mechanical properties and high degradation rates. Nevertheless, recent works [47,48,49] in tissue engineering demonstrate quite a good potential for the use of collagen-based composite fibers. In [24], the authors introduced the bacterial cellulose–collagen nanocomposites, and showed that the increase of bacterial cellulose content led to a better mechanical stability. Particular attention should be paid to bone-mimicking electrospun materials based on collagen and bioactive inorganic inclusions (HAP, tricalcium phosphate (TCP)) that will be discussed further in this study. Among a great variety of natural polymer-natural polymer nanocomposites, chitosan/silk fibroin (CS/SF) and chitosan/gelatin electrospun materials can be highlighted. In [27], Lai et al. fabricated CS/SF (1:1) electrospun mats and studied the morphological features of the materials in sufficient detail. According to the results of alkaline phosphatase activity investigation and Alizarin Red staining, nanocomposite materials turned out to preserve chitosan osteogenic characteristics and increase human bone marrow stem cells differentiation. Park et al. [50] reported the manufacture of electrospun composite fibers using formic acid as a solvent, which contributed to the formation of nanofibers with a narrow diameter range compared to pure silk fibroin fibers. In general, we can conclude that chitosan, as a cationic polyelectrolyte, increases the conductivity of composite solutions and facilitates the molding process via polymeric jet stabilization. In order to induce silk fibroin β-sheet conformation, which in turn leads to the improvement of mechanical properties, the fibers of such composition are usually treated with alcohols [51]. The attempts to apply chitosan/gelatin substrates to BTE revealed that the addition of gelatin to chitosan fibers led to the enhanced hydrophilicity and biodegradation of the materials along with low tensile strength and Young’s modulus values. At the same time, the materials largely supported cell viability promoting the proper cell attachment to the substrate surface [29]. A number of studies have noted potentially successful combination of chitosan with the natural linear polysaccharide agarose that is widely applied in pharmaceutics. The introduction of agarose at a mass content of 30–50% into the molding system contributed to the formation of smooth cylindrical fibers with a controllable fiber thickness and essential biocompatibility. The obvious disadvantage is connected with the use of toxic solvents, such as trifluoroacetic acid or dichlormethane, in relation to chitosan and agarose [32,33]. The properties of chitosan, which are rather attractive for biomedical applications, allow scientists to consider it for the development of controlled-release drug delivery platforms. In particular, the authors [52] have presented a method for obtaining f-CNOs/diclofenac-complex integrated chitosan nanocomposite hydrogels fabricated via ionic gelation. The identified characteristics of such drug delivery system, includes dual pH- and thermo-responsive controlled release at a temperature range of 37–55 °C, essential cell viability against human fibroblasts, as well as uniform particle distribution, pave the way to the design of chitosan-containing nanofibrous composites for drug delivery.

*Synthetic polymers*. Currently, synthetic polymers are significant constituents in numerous fields of life, including the food industry, pharmaceutics, and tissue engineering. Unlike natural polymers with excellent biocompatibility that possess high degradation rates and weak mechanical characteristics, materials based on synthetic polymers can be devoid of mentioned flaws, and hence act as artificial matrices with controllable biodegradation. However, the issues of possible inflammations related to insufficient compatibility with tissues due to the presence of toxic by-products, as well as unsuitable crystallinity and hydrophilicity of polymers still remain unresolved. Today, fibrous materials based on such polymers as poly(l-lactide) (PLLA) [53], poly(lactic-co-glycolic acid) (PLGA) [54], polycaprolactone (PCL) [55], and nylon-6 (N6) [56] are widely performed in the literature sources aimed at the development of BTE. Poly(vinyl alcohol) (PVA) and poly(ethylene oxide) (PEO) are usually added to specified polymers in order to increase hydrophilicity and facilitate fiber formation via electrospinning. In BTE, a lot of research interest has been attracted by N6 due to its high stability in human body and chemical structure resembling collagen-1. Optimized surface properties of electrospun PLLA-based nanofibers in relation to pre-osteoblast MC3T3-E1 cells deposited on fibrous constructions were discussed in [57]. Gao et al. [58] manufactured random and aligned fibrous materials from PCL and decellularized meniscus ECM (DMECM). Next, we will consider synthetic polymer-synthetic polymer composites.

As noted above, N6-consisting fibers are of considerable interest for the study in BTE and related branches of regenerative medicine. Thus, the study [36] demonstrates an approach to N6/PVA-based nanofibers preparation. According to the results, the authors stated that the presence of PVA promoted hydrogen bonding interaction and N6 crystalline conformation change. In addition, the enhanced wettability achieved by insertion of PVA led to a better MC3T3-E1 cell attachment. Xu et al. [37] presented 3D electrospun PCL/PLA nanofibrous scaffolds and observed the effect of PLA content on physical and chemical properties of the ultimate materials. Thus, the increased PLA content was confirmed to improve bioactivity, which, based on the analysis of human mesenchymal stem cells (hMSCs) behavior, resulted in high alkaline phosphatase activity and gene expression level. It is important to admit that scaffolds intended for use in BTE not only have to mimic natural ECM, but also possess appropriate mechanical characteristics. Therefore, a number of electrospun composite materials obtained from solutions of polymers with excellent mechanical properties have been presented. For instance, Park et al. [59] fabricated electrospun materials for BTE on the basis of PEO solutions containing polytetrafluoroethylene (PTFE) dispersions. PTFE possesses a great fracture toughness, but remains highly difficult to conduct the electrospinning. The introduction of PEO increases PTFE spinnability and leads to the formation of durable fibers. In [60], a potential application of forcespun PCL/s-CNOs-based composite nanofibers with pH-responsive release of DOX (up to 99% of DOX release at pH 5) and enhanced tensile strength (3.16 MPa) as biomaterials for tissue engineering and controlled release was discussed. In accordance with the scanning electron microscopy investigation, the authors managed to obtain homogeneous nanofibers with average thickness range of 215–596 nm providing a good cell metabolic activity.

Another significant strategy to produce artificial ECM’s for BTE is related to combining natural polymers with biocompatible synthetic polymers. Thus, it becomes possible to mutually compensate the disadvantages of matrices of different types. For example, poor mechanical characteristics and quick degradation of natural polymer-based constructions can be eliminated by the insertion of synthetic polymer, while the natural polymer contributes to a better assimilation of synthetic polymer in the body. Moreover, one of the components of the hybrid fibrous matrix can be capable of inducing specific conformation changes in another component’s structure, as it was demonstrated by the example of N6/PVA matrix. Synthetic polymers are known to be easily fabricated into certain porous structures, which in turn provide appropriate conditions for cell attachment to the rough surface and further proliferation [61]. Electrospun nanofibrous composites based on various combinations of natural and synthetic polymers have been introduced as artificial ECMs for BTE. Thus, electrospun composites of polycaprolactone/carboxymethyl chitosan (PCL/CMC) [62], gelatin/PCL [31] and gelatin/PEO [30], silk fibroin/PVA [28] and silk fibroin/PLCL-PEO [63], collagen/PVA [64] and collagen/PLGA [26], etc., are widely researched for bone tissue repair. In [65], the authors conducted a comparative characterization on bioactivity of PCL/CMC and PCL/chitosan nanofibrous scaffolds obtained by electrospinning. The cultivation of human osteoblast cells (MG63) proved that cell proliferation occurred more successfully for PCL/CMC samples compared to pure PCL- and PCL/chitosan-based substrates. Additionally, the average PCL/CMC fiber thickness was reduced, and the carboxymethylation process allowed to avoid the formation of undesirable fibers. Lee et al. [25] investigated the impact of pore size on bone regeneration for two types of electrospun PCL/collagen hybrid scaffolds with pore sizes of 200 ± 20 and 300 ± 27 µm. According to cell seeding results, the fibers with lower porosity showed better cell proliferation. Regarding the improvement of mechanical properties through the introduction of synthetic polymer into the system, a large number of works aimed at the fabrication of chitosan/PVA- [34,35] and chitosan/silk/PVA-based [66] electrospun materials for biomedical applications should be noted. Such materials possess high tensile stress values depending on the composition, and PVA-constituent enables the fiber formation from hybrid chitosan-containing solutions, which is a challenging task.

The recent studies on biomaterials for tissue regeneration have demonstrated that the ECM-modified scaffolds fabricated on the basis of various compositions and ratios of bone matrix have a positive effect on bone tissue repair. However, the issues related to the lack of specific cell niche and natural structure features for target tissue still remain unresolved. In order to induce bone regeneration and improve clinical performance, the use of decellularized ECM scaffold obtained in vivo or in vitro was proposed. In vivo obtained decellularized ECM scaffolds, or tissue-derived decellularized ECM scaffolds, provide a native medium containing significant proteins, collagen of type I, and such growth factors, as bone morphogenetic proteins. The ECM scaffolds derived from stem cells and bone cells are considered better mimicking constructions.

Today, a strategy associated with the embedding decellularized ECM (dECM) into synthetic polymer hydrogels is considered rather promising for two main reasons. First of all, the described approach allows to enhance cellular response to synthetic materials. Second, it helps to reduce certain mechanical limitations of dECM. The opportunity to fabricate complex composite constructions mimicking bone structure and promoting endogenous cells to recover bone tissue laid the foundation to the ubiquitous study of synthetic polymer matrices enriched with dECM biochemical cues. The study [67] discusses the effect of cell-derived dECM incorporation into PCL-based solutions on the biochemical properties of bi-layered nanofibrous scaffolds with osteogenic and vascular cues obtained via electrospinning. According to bioactivity investigation in osteoblast cultures, dECM-containing scaffolds exhibited sufficient cell proliferation, Alizarin Red staining, and osteopontin-positive extracellular deposits. The increase of bone growth in femoral defects when implanting the matrices, as well as the improved cortical width, make such composite fibrous materials promising for biomedical applications. Another study of bone-derived ECM-incorporated electrospun PCL nanofibrous scaffolds [68] presented in vitro and in vivo investigations that proved the positive effect of materials microenvironment on rat mesenchymal stem cells attachment, proliferation, and osteogenic differentiation. In order to enhance the biofunctionality of the scaffolds for tissue engineering, Carvalho et al. [69] developed cell-derived ECM electrospun PCL composites obtained from ECM derived from human mesenchymal stem cells and umbilical vein endothelial cells. The authors proved that the materials presented significant cell proliferation when the mechanical characteristics remained analogous to pure PCL matrix.

In this section, we conclude that the majority of the materials under discussion are suitable to provide a sufficient microenvironment for cell growth, and hence can be considered as model artificial ECMs for bone regeneration. The combination of natural and synthetic polymers leads to the improvement of electrospun materials properties, providing not only a suitable composition to support metabolic cell activity, but also a stable fibrous structure formed via electrospinning.

### 2.2. Polymer Materials Incorporated with Inorganic Components

In recent years, the tendency to develop and explore composite materials based on polymer hydrogels filled with inorganic nanoparticles has become rather attractive for two main reasons. First, the insertion of particles that possess high bioactivity, and hence contribute to cell differentiation allows scientists to modify the biological properties of the materials. Additionally, the filling with calcium phosphate phases, which compose the mineral foundation of natural bone tissue, brings it closer to the fabrication of extremely bone-like scaffolds. Another significant goal is associated with the improvement of mechanical characteristics, since BTE requires scaffolds with sufficient mechanical strength. At the same time, it is interesting to obtain both complex 3D structures and electrospun fibrous materials using specific types of 3D-printing and electrospinning, respectively. Now, more attention is being given to hybrid scaffolds based on porous bulk hydrogels reinforced with electrospun fibrous components. In this section, we propose to focus mainly on polymer fibers filled with inorganic inclusions.

The mineral constituents of bone tissue are known to be represented by HAP crystals that have platelet-like shape (50 × 20 × 5 nm) and orient in a certain way to the axis of collagen fibers, and amorphous TCP phase. The ratio between the crystalline and amorphous phases in bone tissue is variable, depending on many factors, such as age and genetic or acquired diseases [70]. For this reason, a significant amount of research was aimed at the fabrication of fibrous polymer matrix filled with synthetic HAP and TCP nanoparticles. Thus, in [71], the authors fabricated HAP incorporated poly(d,l-lactide-co-trimethylene carbonate) (PLMC)-based fibers with shape memory effect intended for bone healing. The obtained fibrous materials were proved to possess high shape memory characteristics at a shape recovery ratio of Rr > 99% while increasing shape recovery force compared to pure PLMC nanofibers. In addition, incorporation of HAP nanoparticles showed a sufficient increase in alkaline phosphatase (ALP) secretion and promoted mineral deposition. In order to fully imitate bone ECM’s composition, the studies [72,73,74,75] presented the electrospun nanocomposite materials based on collagen and HAP. Vozzi et al. [76] investigated the impact of HAP content (10, 20, and 30%) and genipin crosslinking on biological features of collagen/gelatin/genipin/HAP fibrous scaffolds. Thus, the authors managed to achieve high cell proliferation for composite material with 10% HAP content, confirming that the adhesion and colonization of human primary osteoblasts occurred inside the scaffold after day 3. 

**Figure 2:**
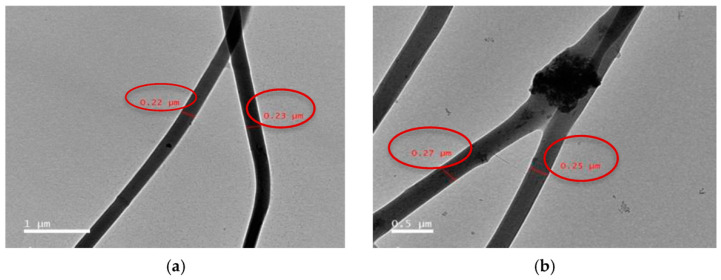
Transmission electron microscopy of prepared fibers: sample CA13 at image scale 1 μm (**a**), HAP particles dispersed inside the sample CA13 at image scale 0.5 μm (**b**) [77].

Recent works in BTE discuss HAP reinforced cellulose nanofibers that were shown to possess appropriate strength and thermostability, as well as good cell viability due to the ultrafine fibrous structure. The study [77] demonstrates the efficacy of HAP-based composites loaded into cellulose acetate nanofibers on osteoblasts and osteoclasts by tacking ALP, osteocalcin, calcium, and total protein concentration. The results of transmission electron microscopy have demonstrated that the authors managed to obtain ultrafine fibers with average diameter of approximately 200 nm and uniform distribution of HAP-based clusters (Figure 2). In [78], the authors used blend electrospinning to produce poly(3-hydroxybutyric acid-co-3-hydrovaleric acid) (PHBV) filled with HAP particles. The composition and structural characteristics, including fiber diameter and surface morphology, were observed by means of scanning electron microscopy (SEM) supported by focused ion beam (FIB) application to confirm the presence of HAP inclusions and its integration with polymer matrix. According to the cell culture results, the hybrid fibrous constructions, obtained without any post-processing, supported cell activity and filopodia formation. Zhang et al. [79] fabricated composite nanofibrous gelatin/β-TCP materials by incorporating 20 wt % β-TCP into the matrix. Compared to pure gelatin-based scaffolds, filled nanofibers promoted the increased cell attachment, and the expression level of Ca-sensing receptor appeared to be much higher.

It should be noted that HAP is considered a compound of a variable composition, for which certain cationic and anionic substitutions (A, B-types) take place. Owing to the ability to provide controllable resorption, carbonate-anion in B-position is believed to be one of the valuable substituents in HAP crystal lattice. Additionally, specific carbonate-containing substances, including carbonate-substituted HAP and calcium carbonates, demonstrated good biocompatibility. This fact opens new paths to develop fibrous ECM-imitating scaffolds filled with calcium carbonates. In a number of studies, the authors do admit favorable features of polymer matrices incorporated with carbonates. In particular, the study [80] describes an approach to fabricate hybrid non-woven fibrous chitosan/gelatin-based mats filled with CaCO_3_ nanoparticles via electrospinning. The investigation of chitosan/gelatin/CaCO_3_ structural features revealed that such composites with carbonate content up to 5 wt % can be successfully electrospun into fibers with a thickness range of 184–237 nm. The increase of mineral filler content resulted in the enhanced antibacterial properties towards *S. aureus*, *C. albicans*, and *B. subtilis*. In [81], a way for obtaining PVA-based fibers filled with HAP, brushite (CaHPO_4_·2H_2_O), and/or calcite particles by “bottom-up” electrospinning technique is proposed. Composite fiber with a diameter of 190–530 nm incorporated with both HAP and calcite phases showed a suitable microenvironment for dental pulp stem cells metabolic activity according to MTT-test. Although the obtained PVA-based fibrous materials are characterized by a high tendency to degrade in neutral medium, they serve as suitable substrates supporting vital cell activity.

**Figure 3:**
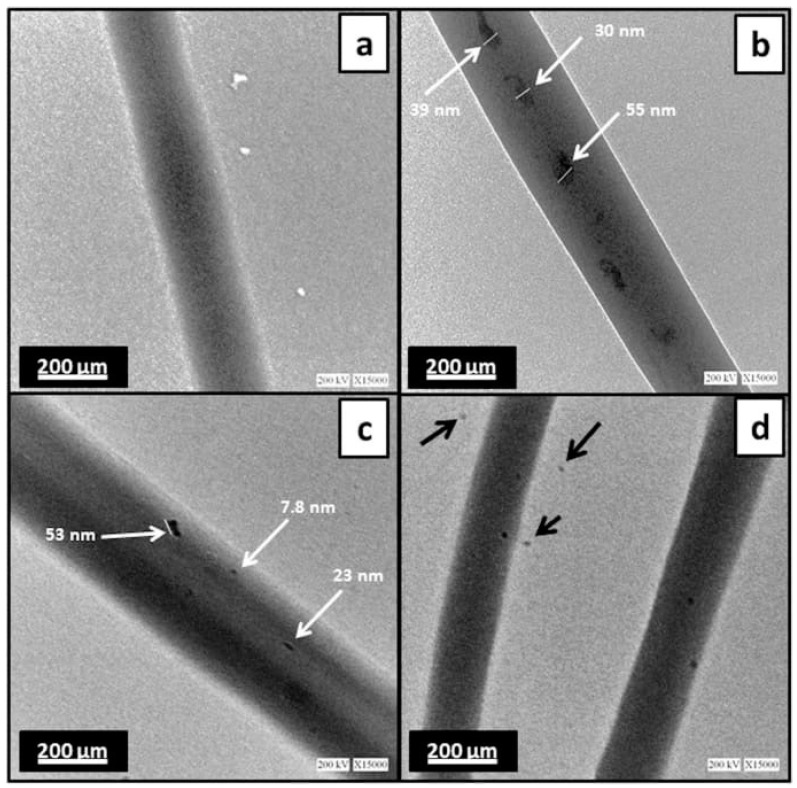
Transmission electron microscopy images of (**a**) PCL, (**b**) PCL/BG, and (**c**,**d**) PCL/gelatin/BG. White arrows indicate nanoparticles within fibers. Black arrows indicate nanoparticles dispersed onto the copper grid [84].

Along with calcium phosphates represented mainly by HAP and TCP, and carbonates, several studies discuss some other components as bioactive fillers. Thus, Nagarajan et al. [82] proposed to reinforce crosslinked gelatin-based matrix by boron nitride in order to increase Young’s modulus without harmful effect on human bone cell activity. In [83], the authors used wet spinning for chitosan–tripolyphosphate (TPP) fibers manufacturing. A better biodegradation of the fibers was indicated due to the lowered crystallinity of chitosan crosslinked by TPP. Moreover, bioactive glass (BG) nanoparticles in the system SiO_2_–CaO–Na_2_O–P_2_O_5_ deserve special attention. In study [84], the authors report a successful application of electrospinning technique to the formation of nanofibrous bilayer scaffold obtained on the basis of PCL/gelatin matrix filled with BG (composition of 45% silica, 25% CaO, 25% Na_2_O, and 5% P_2_O_5_) nanoparticles with a lateral size less than 10 nm previously manufactured by the sol-gel method (Figure 3). According to the analyses, BG incorporations promoted the formation of HAP-like layer with a Ca/P molar ratio of 1.4 after 14 days of immersion is SBF. The BG-reinforced tensile strength of the composite was approximately four times higher than that of filler-free PCL/gelatin matrix.

In summary, various components have been studied for the role of inorganic inclusions. However, the tendency to incorporate the artificial ECM with phosphate nanoparticles is traced to the greatest extent. Therefore, HAP, β-TCP, and TPP inclusions are highly applicable in bone tissue engineering, and, hence, can be recommended for the electrospun materials reinforcement in order to improve mechanical features and natural tissue formation. In addition to this, the list of compounds to be placed in the artificial ECM can be expanded by other biocompatible inclusions, such as calcium carbonate, boron nitride, BG nanoparticles, etc., that are capable of supporting cell activity.

## 3. Application of Electrospinning Techniques for Fibrous Scaffold Fabrication

Biomimicking approach in tissue engineering is aimed at the development of the artificial ECM that meets all the necessary structural features characteristic of the natural matrix. Bone tissue engineering is not an exception to that, and follows the trend of manufacturing a bone ECM-like material. Since the natural ECM consists of collagen fibers oriented in a certain way, the search for methods of fibrous scaffold fabrication is an exceedingly important task. In addition, the scaffold microenvironment should promote osteoinduction, and provide cell attachment due to appropriate surface morphology and roughness.

There exist a number of techniques for fiber manufacturing, such as nanolithography, blow spinning, fibrillation melt, 3D-printing, and self-assembly. However, due to certain advantages and technological features, electrospinning techniques attracted greatest interest when developing fibrous matrices.

### 3.1. Electrospinning Principle

Various techniques of electrospinning pave the way to the obtaining of nanoscale fibrous materials with high surface to volume ratio and controllable fiber thickness that can be used for specific biomedical purposes, including bioscaffolding in tissue engineering. Table 2 demonstrates the most complete classification of techniques performed in the literature. The main principle of nanofiber production via electrospinning is related to the spraying and stretching polymer solutions or melts in the electrostatic field caused by high voltage application [85]. The electrostatic field generated in the working chamber of the electrospinning unit induces the formation of thin jets from composite molding solution. As a rule, one of the electrodes located in the chamber is in contact with a droplet of the sample, while another electrode serves as a collector for solidified fiber deposition. The effect of the electrostatic field on the polymer solution droplet plays the key role, since it causes the droplet deformation, that is, the stretching to an unstable jet, which then continue to stretch, thin, and bend repeatedly as the solvent evaporates. The fibers solidified as a result of solvent evaporation lay on the collecting electrode. In more detail, the stages of the traditional electrospinning process including the Taylor cone formation stage are described in [86,87]. It is important to note that the droplet deformation and subsequent stretching into the jet occurs when the force of the electrostatic field begins to exceed the surface tension force for the droplet. Thus, the surface tension, among others, is a significant parameter of the process. The voltage source provides voltage values up to hundreds of kilovolts. Such a voltage applied to the polymer solution or melt induces surface electric charges that, owing to Coulomb repulsion, contribute to the jet formation.

**Figure 4:**
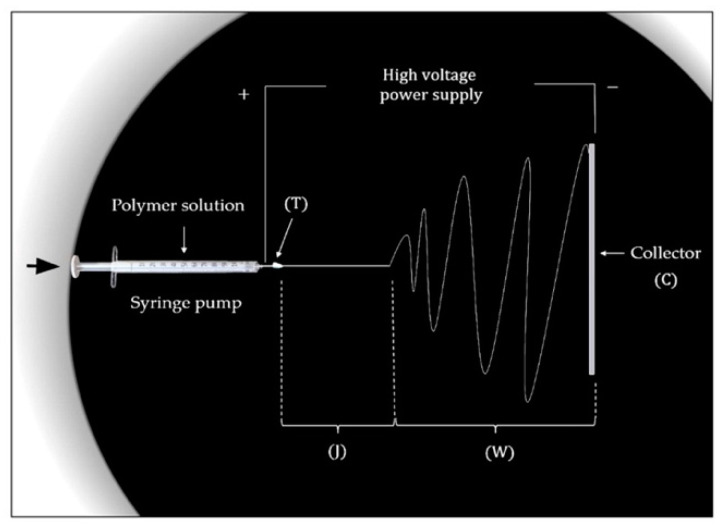
Schematic view of the electrospinning equipment with the four distinct states: Taylor cone formation area (T); stable-straight jet part (J); unstable, whipping region (W); fibers collection phase (C) [87].

**Figure 5:**
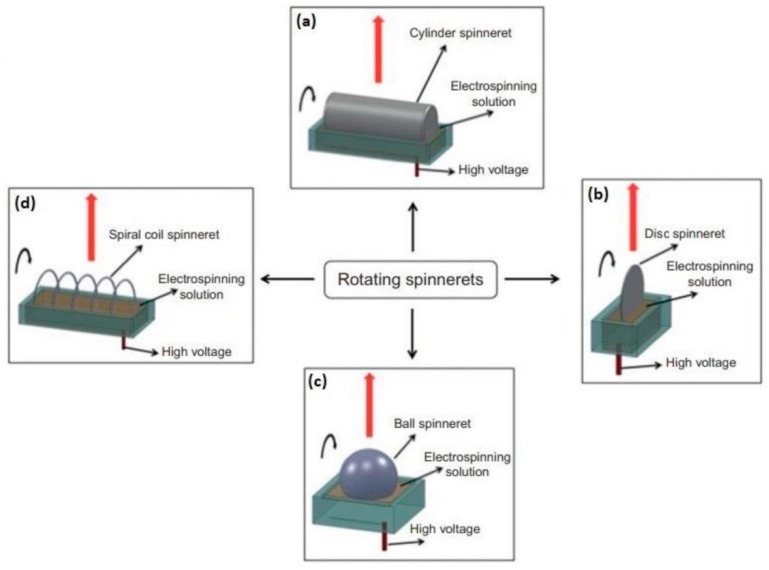
Schematic diagram depicting the rotating spinnerets: cylindric spinneret (**a**), disc spinneret (**b**), ball spineret (**c**), and wire/coil spinneret (**d**) in needleless electrospinning (upward electrospinning direction along the red arrow) [90].

According to the proposed classification, electrospinning techniques can be divided into two large groups—needle-based and needleless electrospinning [88]. The typical unit for the implementation of needle-based electrospinning consists of four main parts: a rotator with a syringe pump, a metal syringe needle (or capillary) in contact with the polymer droplet, a high voltage source that generates the electrical potential difference between the electrodes, and a ground collector intended for fiber deposition. The conventional scheme of needle-based electrospinning unit is shown in Figure 4. Currently, along with needle-based electrospinning techniques, needleless, or capillary-free, electrospinning types, such as two-layer fluid electrospinning, melt differential electrospinning, and rotating roller electrospinning, are widely in demand [89]. In this case, the working chambers do not contain capillary nozzles, and provide the upward movement of polymer jets. In this regard, the collector is usually located in the upper part of the chamber, while a rotating cylindrical electrode with the droplet is installed at the bottom. The discussed unit scheme is implemented for rotating roller electrospinning using Nanospider technology (Figure 5).

Many studies on electrospinning note a number of parameters that defines the success of the electrospinning process. The stated parameters include the structure and molecular weight of the polymer, concentration and viscosity of the solution (the latter is directly affected by molecular weight), its conductivity and dielectric constant value, voltage value and the distance between the electrodes, the properties of the solvent, including viscosity and surface tension, and air humidity.

Along with Nanospider technology used in needleless electrospinning, there exists a number of needle-based techniques that have been performed in recent research papers. These techniques include melt, coaxial, modified collector, solution, and modified bubble electrospinning. Thus, melt electrospinning allows to obtain non-woven materials with a diameter range of approximately 270–500 nm by cooling liquid polymer melt, and, hence, removing the residual amounts of liquid [91]. Coaxial electrospinning type successfully applied to such hydrophilic compounds as poly(ethylene glycol) (PEG) and collagen, has permitted the fabrication of fibers with core/shell structure [92]. The application of modified collector electrospinning in BTE has revealed a way to mimic the orientation of collagen nanofibers in the ECM. This mimicking method is considered rather essential for producing polymer-based nanofibers with anisotropic alignment, since such a structure promotes the cell adhesion. Moreover, in [93], the nanofiber alignment was proved to ensure a higher stiffness and elastic modulus (up to 19.7 MPa) of PCL-based nanofibers. The solution electrospinning technique laid the foundation to the obtaining ethacrylat fibrous material for a better cell infiltration. Thus, Nam et al. [94] managed to obtain ethacrylat PCL scaffolds using sodium chloride (NaCl) as a porogen. Such method allowed the cells to migrate to a depth of several mm into the scaffold. Compared to conventional electrospinning techniques, modified bubble electrospinning (MBE) involves higher voltage values to produce nanofibers with the improved structural features. MBE has demonstrated a moderate success in relation to silk fibroin nanofibers [95].

### 3.2. Advantages and Key Issues of Electrospinning

Despite the inability to manufacture fibrous materials with complex structures, including the fibrous materials with homogeneous pore distribution, electrospinning remains a simple and low-cost method to obtain nanofibrous materials holding a set of structural and biological properties valuable for tissue engineering.

The literature provides a tremendous number of studies on the use of the electrospinning techniques for obtaining nanofibrous materials based on both natural and synthetic polymers, as well as their combinations. The main task of developing the fibrous scaffolds that are architecturally analogous to the native bone ECM is related to the stabilization of the polymer jet by optimizing certain conditions. Thus, as it was mentioned in this study, the solution properties, unit parameters, and environmental conditions of the chamber are exceedingly determinative. Regarding the solution properties, which are optimized at the stage preceding the electrospinning process, nanofibrous materials are usually obtained from polymer solutions with a polymer concentration of less than 20 wt %, surface tension not exceeding 50 mN/m, and dynamic viscosity values of ~1 Pa·s. At the same time, the boiling point of the solvent should not be too low to avoid its excessively rapid evaporation and further jet solidification at the forming stage. Based on the analysis of the literature, particular attention is paid to the viscosity of pure polymer solutions and composite polymer gels incorporated with inorganic components or crosslinking agents. Thus, the reduced viscosity values may cause the spreading of the droplet over the electrode surface (for needleless electrospinning type), and the high viscosity values exceeding a threshold value do not quench capillary waves destroying the polymer jet. Another challenging task related to the fabrication of fibers based on natural polymers concerns the solvent selection. Due to limited solubility in organic solvents that is explained by a strong polarity or specific crystal structure, natural polymers are usually dissolved in aqueous media that are difficult to remove during the molding process. Strong hydrogen bonds with water molecules, as well as high mass values of natural polymers lead to the increased viscosity of the solutions at relatively low concentration levels.

Among a sufficient number of practical limitations when using electrospinning for BTE, the authors admit the shortcomings of the fabricated materials, such as the reduced cell infiltration and a lack of mechanical strength. The pore sizes in the electrospun fibrous materials are significantly lower than the standard pore size range (100–500 µm) of natural bone tissue, which limits the cell infiltration. In [96], the prevention of vascular growth in in vivo experiments was demonstrated and explained by the presence of small pores, which in turn resulted in the decreased transport of nutritious substances and waste removal. As it was noted, the mechanical characteristics that are influenced by biomaterial composition, nanofiber thickness, porosity, and orientation, do not always meet the requirements of the potential fibrous scaffold. Possible strategies to eliminate some of the flaws associated with mechanical properties of the electrospun materials for BTE, including the blending with inorganic phase and thermal treatment, were discussed in [86].

It should be noted that the majority of research papers devoted to the development and functionalization of non-woven polymer materials for biomedical applications discuss forcespinning as more preferable technique in comparison with electrospinning. In this method called forcespinning, the electrostatic field is replaced with centrifugal forces, which in combination with multiple configurations of interchangeable spinnerets allows to overcome specific limitations typical for electrospinning, including high electric fields and strictly defined solution characteristics. Thus, the selection of solution systems to be spun into nanofibers can be expanded by non-conductive polymer solutions. In addition, the use of high temperature solvents becomes acceptable in this method. For instance, the work [97] discusses pH-responsive poly 4-hydroxyphenyl ethacrylate single-walled carbon nanotube nanocomposite fibrous membranes for drug delivery obtained via forcespinning followed by a heat pressure technique. The favorable mechanical characteristics, such as the essential tensile strength (13.7 ± 3.2 GPa) and Young’s modulus (243.3 ± 5.2 GPa), represent the prepared material as one of the stiffest nanocomposites among all the reported to date. Along with high cell viability of the membranes functionalized with hydrophobic molecules of curcumin, the materials exhibited excellent adsorption capability towards Pb^2+^ and Cd^2+^ ions, which in turn opens an opportunity to apply them in the field of environmental remediation. In [98], Mamidi et al. introduced an approach to the production of fiber-aligned curcumin-embedded scaffolds based on gelatin/PLA composites by using forcespinning technique. The materials under discussion were shown to support the growth of human fibroblast cells and the sustained curcumin release over 15 days.

The mentioned technology limitations and research challenges, however, cannot belittle the versatility and efficiency of the method towards fabrication of polymer fibers based on various phase compositions. Nanofibrous composites prepared via electrospinning were shown to possess high specific surface area, semipermeability, and structural features promoting the cell adhesion. Therefore, their further study and improvement are crucial directions in BTE.

## 4. Conclusions

The analysis of modern functional materials intended for bone tissue recovery has demonstrated that the electrospun fibrous constructions have gained a considerable interest from the research community. Owing to their structural features, including high surface to volume ratio, tunable fiber thickness, and surface roughness appropriate for cell attachment among others, nanofibrous scaffolds can properly replicate the native collagen-based ECM fibrillar structure. Among a variety of methods to manufacture fibrous constructions, electrospinning is believed to be the most applicable due to its versatility, simplicity, high fiber production rates, and, hence, potential general availability. However, obvious advantages of the method do not exclude a number of technological obstacles when applying it to nanofibrous bioscaffolds fabrication. In particular, many physiochemical characteristics of the molding solution, including viscosity and solvent properties, should be strictly optimized to achieve a stable fiber formation process. Moreover, the nature of certain polymers, such as chitosan, does not allow to produce stabilized jets from molding polymer solutions in the absence of biologically non-friendly acidic components. On the basis of a tremendous number of studies on natural and synthetic polymers, it can be concluded that the combining of various natural and synthetic polymers is a promising strategy to improve biological and mechanical properties of the artificial matrix. Thus, composite PCL/collagen-, silk fibroin/chitosan-, N6/PVA-, and PCL/PLA-based electrospun materials were proved to find a successful application in BTE. Additionally, a strategy to fabricate hybrid nanofibrous polymer matrices incorporated with bioactive inorganic particles is also promising for the improvement of biomaterial features. Thus, the list of potentially applicable mineral constituents limited until recently by HAP and TCP that occurs in natural bone tissue can be expanded by calcium carbonates, bioactive glass nanoparticles, and boron nitride.

## 5. Future Perspectives

Nanofibrous artificial matrices fabricated via various electrospinning techniques do act as suitable substrates with favorable surface morphology that are able to maintain vital metabolic cell activity. However, the majority of the electrospun scaffolds possess poor tensile strength characteristics, and undergo relatively rapid degradation in neutral microenvironments, which, as a rule, can be eliminated by the introduction of biologically non-friendly crosslinking agents causing an immunological response. Although this brief review suggests focusing on unmodified polymer materials and does not pay a sufficient attention to the crosslinked polymer composites, further research could be aimed at the overview and classification of the modern electrospun crosslinked polymer scaffolds with optimal compositions and reduced effect of crosslinking agents on cell viability. In addition, any research in the field of bioscaffolding, involving composites of natural, synthetic, and crosslinked polymers, as well as mineral-incorporated composites, requires more in vivo studies. Moreover, it is important to admit that the fibers fabricated via electrospinning techniques do not always seem convenient for further implantation or soaking in testing media. At that, there is a number of technological shortcomings associated with the separation of deposited fibers from the collecting electrode. Thus, in order to obtain the constructions combining the advanced features of nanofibrous and 3D scaffolds with complex structure, we propose to pay more attention to the joint electrospinning and 3D-printing technologies and other mixed techniques providing a controllable 3D design.

## Figures and Tables

**Table 1:** Modern natural and synthetic polymers for BTE.

Polymer Type		Suitable Composite	Feature
**Natural polymers**	Collagen	collagen/cellulose [24], PCL/collagen [25], collagen/PLGA [26]	Significant component of native ECM, low cytotoxic response, weak mechanical properties, high degradation rate
	Silk fibroin	silk fibroin/chitosan [27], silk fibroin/PVA, silk fibroin/PLA, silk fibroin/PEO [28]	Sufficient biocompatibility, strong mechanical properties, low degradation, easy to process, no immunogenic response in vivo
	Gelatin	chitosan/gelatin [29], gelatin/PEO [30], gelatin/PCL [31], gelatin/silk fibroin [15]	Similar to collagen in structure, relatively high tensile modulus, suitable biocompatibility, highly affordable
	Chitosan	silk fibroin/chitosan [27], chitosan/gelatin [29], chitosan/agarose [32,33], chitosan/PVA [34,35], chitosan/PEO [35]	Suitable biocompatibility, strong fibers in combination with PVA, requires toxic acidic agents for electrospinning
	Cellulose	collagen/cellulose [24]	Significant biocompatibility, weak mechanical properties, high degradation rate
**Synthetic polymers**	N6	N6/PVA [36]	Sufficient biocompatibility, controllable conformation, enhanced wettability resulting in good MC3T3-E1 cell attachment for N6/PVA
	PCL	PCL/collagen [25], PCL/PLA [37]	Sufficient biocompatibility and biodegradability, highly affordable, increased hydrophobicity resulting in poor cell attachment
	PLA	PCL/PLA [37]	Sufficient biocompatibility, improved mechanical properties compare to analogs, low degradation, inflammatory reactions caused by its by-product
	PLGA	collagen/PLGA [26]	Sufficient biocompatibility, high degradation rate compared to PLA
	PEO	gelatin/PEO [30], silk fibroin/PEO [28], chitosan/PEO [35]	Sufficient biocompatibility, mainly used as additive to improve properties of the artificial ECM
	PVA	silk fibroin/PVA [28], chitosan/PVA [34,35], N6/PVA [36]	Suitable biocompatibility, mainly used as additive to improve properties of the artificial ECM, highly affordable, process with various hydrolysis degrees, high degradation rate

**Table 2:** Electrospinning techniques.

**Electrospinning type**	**Needle-based (capillary)**	Multiaxial electrospinning: -coaxial -triaxial
		Bi-component electrospinning
		Multineddle electrospinning
		Electroblowing/Gas-assisted/Gas jet electrospinning
		Magnetic field assisted electrospinning
		Conjugate electrospinning
		Centrifugal electrospinning
	**Needleless (capillary-free)**	Bubble electrospinning
		Two-layer fluid electrospinning
		Splashing electrospinning
		Melt differential electrospinning
		Gas-assisted melt differential electrospinning
		Rotary cone electrospinning
		Rotating roller electrospinning/Nanospider technology
		Edge electrospinning
		Blown bubble electrospinning

## Data Availability

All the data is available within the manuscript.
